# Anti-Inflammasome Effect of Impressic Acid on Diesel Exhaust Particulate Matter-Induced NLRP1 Inflammasome via the Keap1/p62/Nrf2-Signaling Pathway in Keratinocytes

**DOI:** 10.3390/antiox14050610

**Published:** 2025-05-19

**Authors:** Seung Yeon Lee, Gi Ho Lee, Jeonghwan Maeng, Su Yeon Kim, Hwi-Yeol Yun, Gil-Saeng Jeong, Hye Gwang Jeong

**Affiliations:** College of Pharmacy, Chungnam National University, Daejeon 34134, Republic of Korea; sy9842@o.cnu.ac.kr (S.Y.L.); ghk1900@cnu.ac.kr (G.H.L.); jhmaeng21@o.cnu.ac.kr (J.M.); tndusl0215@o.cnu.ac.kr (S.Y.K.); hyyun@cnu.ac.kr (H.-Y.Y.); gsjeong@cnu.ac.kr (G.-S.J.)

**Keywords:** diesel exhaust particles, NLRP1 inflammasome, impressic acid, Nrf2, keratinocytes

## Abstract

Diesel exhaust particulate (DEP) is widely recognized to weaken lung function and skin diseases. When the skin, which defends against external factors, is exposed to PM2.5, various chronic inflammatory diseases occur. When keratinocytes recognize harmful signals, they synthesize the NOD-like receptor protein 1 (NLRP1) inflammasome. DEP enhances NF-κB signaling and NLRP1 inflammasome expression through the interaction of TXNIP with NLRP1 in keratinocytes. Although many studies have reported the anti-inflammatory and antioxidant characteristics of Impressic acid (IPA), the umbrella consequences of IPA for PM2.5-influenced inflammasomes and the associated mechanisms remain unknown. Therefore, this study aimed to examine the protective function of IPA against inflammation in human keratinocytes. IPA attenuated the NLRP1 expression, caspase-1, IL-1β actuation, and NF-κB and IκB phosphorylation induction by DEP. IPA upregulated the Nrf2, HO-1, and NQO1 expression through CaMKKβ, AMPK, and GSK3β phosphorylation. Also, IPA led to the elevation of p62 and the degradation of the Keap1 protein. ML385 reversed the suppressive effect of IPA on the NLRP1 inflammasome, which was enhanced by DEP, and NAC counteracted the effect of ML385. These findings indicate that IPA can suppress inflammation induced by PM2.5 by expressing antioxidant enzymes through the Keap1/p62/Nrf2-signaling pathway in human keratinocytes.

## 1. Introduction

Diesel exhaust particles (DEP), which are complexes of gas and particulate compounds generated by incomplete diesel fuel combustion, constitute a large part of fine particulate matter (PM2.5) and are associated with various health risks [1]. PM2.5, characterized by an aerodynamic diameter of 2.5 mm or less, is more likely to transport toxic substances and exhibit extended suspension in the atmosphere [2]. PM2.5, which enters the human body and induces oxidative stress and various diseases, serves as a significant atmospheric air pollution indicator through various activities [3,4]. Continuous exposure to PM2.5 causes inflammation. Chronic exposure to PM2.5 can cause inflammation, impair lung function, and allow harmful particles to enter the bloodstream and skin, thereby increasing the risk of cardiovascular diseases such as hypertension and heart disease as well as skin-related illness [5].

As the principal and most extensive organ of the human body, the skin functions as a physical obstruction, constituting the foremost line of protection against external influences [6]. It reduces the penetration of chemicals, protects against microorganisms, and penetration of exogenous substances such as microbial infection and damage affects the risk of inflammatory diseases and induces immune responses [7]. Particulate matter, specifically PM2.5, can penetrate both the compromised and intact skin barriers, potentially leading to systemic inflammatory reactions and skin damage [8]. Inflammatory processes induced by exposure to PM2.5 are linked to various inflammatory conditions, such as atopic dermatitis, arteriosclerosis, and chronic bronchitis [9,10]. Upon recognizing noxious signals in the human body, keratinocytes begin synthesizing inflammatory cytokines and inflammasome complexes, especially NOD-like receptor protein 1 (NLRP1) inflammasomes, which mediate skin inflammation and have significant implications for skin health and disease [11,12]. Inflammasomes are frequently located in immune cells such as macrophages, monocytes, and keratinocytes, representing a category of intracellular multiprotein composites that play crucial roles in regulating immune reactions [13,14,15]. NLRP1 is a newly identified inflammasome integral to the innate immune system that recognizes pathogens and initiates inflammatory responses [16]. NLRP1 has been shown to rely on the adaptor protein ASC associated with the C-terminal caspase activation and recruitment domain (CARD), which is characterized by two death-fold domains, the pyrin domain (PYD) and the CARD, which are essential for interleukin-1 (IL-1) secretion and interact with (pro-)caspase-1 to promote protease dimerization [17,18,19,20]. When NLRP1 binds to ASC and procaspase-1, an inflammasome is assembled and caspase-1 is activated through autocatalytic cleavage [21]. Caspase-1 functions as an influencing molecule within inflammasomes and is a cysteine protease family member that regulates cell death and apoptosis [22]. Activated caspase-1 facilitates the processing and enhancement of the pro-inflammatory cytokines pro-IL-1β and pro-IL-18, promoting inflammatory responses [23,24]. It is noteworthy that caspase-1 activity is essential both for pro-IL-1β and pro-IL-18 activation and for their subsequent excretion and liberation [25]. Gasdermin D (GSDMD) is cleaved by caspase-1, generating an amino-terminal fragment that subsequently integrates into the extracellular membrane via oligomerization [26]. Cleaved GSDMD translocates from the cytosol to the lipid bilayer, where it accumulates in the pores that trigger pyroptosis and release IL-1β and IL-18 into the extracellular environment [27]. Pro-inflammatory cytokines, specifically pro-IL-1β, pro-IL-18, and GSDMD, serve as substrates for caspase-1 [28,29]. The inflammatory response is intricately linked to oxidative stress, whereby an increase in reactive oxygen species (ROS) facilitates inflammatory cell migration and contributes to skin tissue [30].

Impressic acid (IPA) is a lupane-type triterpenoid isolated from the plant species *Acanthopanax Koreanum* [31]. IPA has various pharmacological activities, including protection against vascular endothelial dysfunction and inflammatory diseases. Several studies have reported its anti-inflammatory, antioxidant, and anticancer effects, and it has been used therapeutically for the healing of rheumatism, type 2 diabetes, hepatitis, and inflammatory disorders [32,33]. Also, IPA is known to prevent cartilage degradation disorders [34] and inhibit the NF-κB activation induced by Tumor Necrosis Factor-alpha (TNF-α) [35]. Recently, IPA has been documented to reduce the inflammatory replication elicited by LPS in RAW264.7 macrophages [33]. Despite the various important biological properties of IPA, the defensive role of IPA in contrast to the PM2.5-induced inflammasome and its molecular mechanism has not been established. Therefore, this study aimed to examine the protective cue of IPA against the activation of inflammasome induced by PM2.5 in human keratinocyte HaCaT cells.

## 2. Materials and Methods

### 2.1. Chemicals and Reagents

The spontaneously immortalized human keratinocyte cell line HaCaT was purchased from CLS Cell Lines Service GmbH (Eppelheim, Baden-Württemberg, Germany) and utilized. DMEM, penicillin-streptomycin, trypsin, and FBS were obtained from Welgene (Gyeongsan, Republic of Korea). IPA was supplied by Professor Young Ho Kim (Chungnam National University, Daejeon, Republic of Korea), and the IPA structure is illustrated in Figure S1A. Diesel particulate matter (SRM 2975, National Institute of Standards and Technology, Gaithersburg, MD, USA) was used as a reference material [36], 3-(4,5-dimethylthiazol-2-yl)-2,5-diphenyltetrazolium bromide (MTT), LY294002, STO-609, ML385, Chloroquine, and dimethyl sulfoxide (DMSO) were obtained from Sigma-Aldrich (St. Louis, MO, USA). A lactate dehydrogenase (LDH) assay kit was purchased from Roche Applied Science (Indianapolis, IN, USA). Compound C and NAC were purchased from Tocris (Cookson, Bristol, UK). W7 was acquired from Calbiochem (LA Jolla, CA, USA) and EDTA was obtained from GenDEPOT (Barker, TX, USA). Antibodies against NLRP1, Cleaved caspase-1, Cleaved IL-1β, p-NF-κB, NF-κB, p-IκB, IκB, TXNIP, p-Akt, p-CaMKKβ, p-AMPK, p-GSK3β, GCLC, p62, LC3B, and secondary antibodies against HRP-linked anti-mouse or anti-rabbit IgG were purchased from Cell Signaling Technology (Beverly, MA, USA). Antibodies against ASC, NEK7, Nrf2, HO-1, NQO1, and β-actin were purchased from Santa Cruz Biotechnology (Dallas, TX, USA). The N-terminal GSDMD and p-Nrf2 antibodies were obtained from Abcam (Cambridge, MA, US). Keap1 and lamin B1 were purchased from Bioss Antibody, Inc. (Woburn, MA, USA). All kits were used per the manufacturer’s guidelines, and all other chemicals used in the present study were of the highest purity available commercially.

### 2.2. Cell Culture and Treatment

HaCaT cells were cultured with DMEM enriched with 10% FBS and 1% penicillin and streptomycin at 37 °C and 5% CO_2_ in a humidified incubator. When the cells were approximately 80% confluent, they were subjected to a 12 h pretreatment with IPA, followed by a 24 h treatment with DEP. DEP was prepared as a 1 mg/mL stock concentration in sterile distilled water and sonicated with 3 s pulses (3 s ON, 10 s OFF) for a total duration of 2 min to ensure proper dispersion before application to the cells.

### 2.3. Cell Viability and Cytotoxicity Assay

MTT and LDH assays were performed to confirm the viability and cytotoxicity of IPA in HaCaT cells. HaCaT cells (1 × 10^5^ cells/well) were seeded in 48-well plates and let them rest for 24 h. Next, the cells were used with different amounts of IPA (1–20 μM) and exposed to DEP 100 μg/mL for a whole day. The MTT assay was used to evaluate cell viability. After emptying the medium, MTT solution (final concentration, 1 mg/mL) was added to each well for 30 min. The formazan crystals formed were mixed with DMSO, and the absorbance was determined using a BioTek Synergy HT microplate reader (BioTek Instruments, Winooski, VT, USA) at 550 nm. A lactate dehydrogenase (LDH) assay was used to assess cytotoxicity. The medium was collected and combined with the LDH liquid. The absorbance was recorded at 490 nm using a microplate reader. Percentages of cell survival and death were calculated by comparing the absorption measurements of the samples with those of the control group treated with DMSO alone. Treatment of the cells with varying concentrations of IPA (1–20 μM) for 24 h demonstrated a release in cell viability and an augmentation in cytotoxicity at the concentration of 20 μM (Figure S1B). IPA Pretreatment recovered the cell viability decreased by DEP and reduced the cytotoxicity induced by DEP in a concentration-dependent manner (Figure S1C).

### 2.4. Western Blot Analysis

The HaCaT cells were collected and lysed in CETi lysis buffer (TransLab, Daejeon, Republic of Korea). The cells were pelleted by centrifugation at 13,000 rpm for 15 min, and the supernatant was obtained as the sample source. The protein samples were quantified at 595 nm using a protein assay kit (Pro-Measure, Intron Biotechnology, Seongnam, Republic of Korea), and equal amounts of total cellular protein were boiled for 5 min. The proteins were electrophoresed on a 10% sodium dodecyl sulfate-polyacrylamide gel and transferred onto nitrocellulose membranes. Following a 1 h blocking period using 5% skim milk, the membranes were incubated overnight with linear antibodies and then incubated with secondary antibodies. Protein bands were measured using the Enhanced HiSol ECL Plus Detection Kit (BioFact, Daejeon, Republic of Korea). Band intensities from all western blot images were quantified using the ImageJ (Version 1.54p) program, and the quantified band intensities are provided in Figures S2–S7.

### 2.5. Real-Time PCR

Total RNA was isolated from HaCaT cells using RNAiso Plus (total RNA extraction reagent; Takara, Shiga, Japan), and cDNA was synthesized using the BioFact RT Series Kit. RT-PCR was continuously performed using the Bio-Rad CFX Connect Real-Time PCR software, version 1.4.1 (Bio-Rad Laboratories, Hercules, CA, USA). The specific primers utilized in the study are detailed below: NLRP1 (NM_033004.4) Forward, 5′-ATTGAGGGCAGGCAGCACAGA-3′; NLRP1 (NM_033004.4) Reverse, 5′-CTCCTTCAGGTTTCTGGTGACC-3′; HO-1 (NM_002133.3) Forward, 5′-CAGCATGCCCCAGGATTTG-3′; HO-1 (NM_002133.3) Reverse, 5′-AGCTGGATGTTGAGCAGGA-3′; NQO1 (NM_000903.3) Forward, 5′-CCTGCCATTCTGAAAGGCTGGT-3′; NQO1 (NM_000903.3) Reverse, 5′-GTGGTGATGGAAAGCACTGCCT-3′; GAPDH (NM_001357943.2) Forward, 5′-GAAGGTGAAGGTCGGAGTCAA-3′; and GAPDH (NM_001357943.2) Reverse, 5′-CTTCCCGTTCTCAGCCATGTA-3.’ The expression values were normalized to those of GAPDH.

### 2.6. Immunoprecipitation Assay

HaCaT cells were cultured and lysed in an immunoprecipitation (IP) buffer. Protein G beads and primary antibodies were then added and incubated at room temperature for 4 h. After washing twice with PBS, the cell lysates (300–500 μg protein) were incubated with reacted primary antibody overnight at 4 °C. The following day, immune complexes were washed twice with PBS and prepared in SDS sample buffer for western blot analysis.

### 2.7. Intracellular ROS Production

Intracellular ROS levels in HaCaT cells were detected using the redox-sensitive fluorescent dye H2DCFDA. The cells were cultured in 48-well plates and treated with IPA and DEP. Following treatment, the upper medium in a single well was removed, and the cells were incubated with 2 μM H2DCFDA at 37 °C for 30 min and then washed twice with PBS. Fluorescence intensity, which serves as an ROS level indicator, was quantified using a fluorescence spectrophotometer at excitation and emission wavelengths of 490 and 530 nm, respectively. Fluorescence signal was normalized to the control group. A negative (NA) and a positive control (DEP 100 µg/mL) were used to verify probe responsiveness and signal specificity.

### 2.8. Statistical Analysis

The trials were practiced in a minimum of three instances (*n* = 3), and the consequences are marked as the mean ± SD derived from separate experiments. Data normality was assessed using the Shapiro–Wilk test prior to conducting ANOVA. Statistical evaluation of the results was performed using one-way ANOVA and the Tukey–Kramer test was used for comparisons among multiple groups. Statistical significance was set at *p* values < 0.01.

## 3. Results

### 3.1. DEP Induces NLRP1 Inflammasome Complex in HaCaT Cells

**Figure 1:**
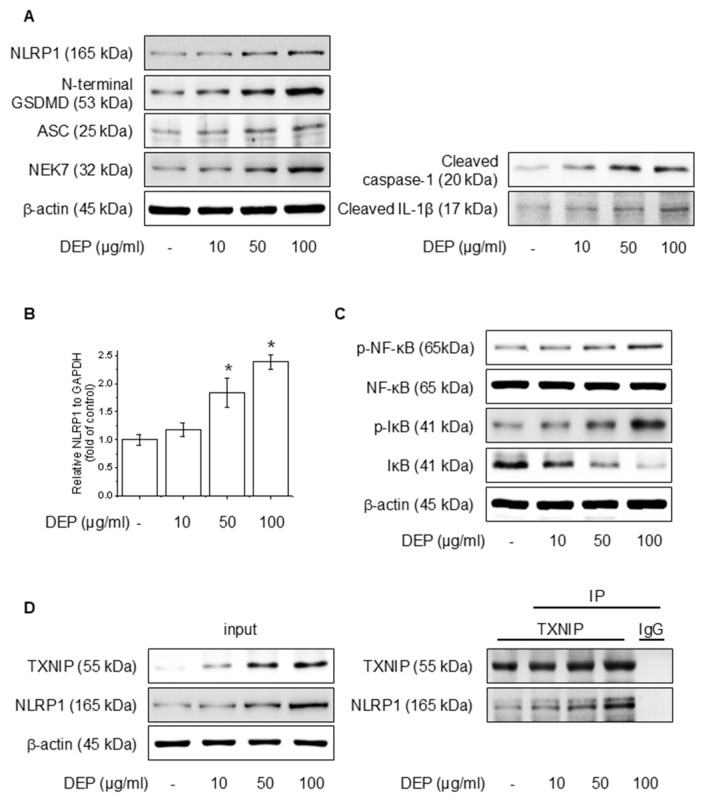
Effect of DEP on NLRP1 inflammasome complex in HaCaT cells. The cells were treated with 10–100 μg/mL DEP for 24 h or 30 min. (**A**) The NLRP1 inflammasome complex was evaluated through western blot, and the (**B**) NLRP1 mRNA level was quantified by RT-PCR. (**C**) The NF-κB and IκB phosphorylation protein levels were assessed via western blot. (**D**) After treatment of DEP for 24 h, the cell lysates were immunoblotted with the specified antibodies. All experiments were performed thrice (*n* = 3). The data are expressed as the mean ± SD. * *p* < 0.01 compared to the control group.

We evaluated the effects of DEP on NLRP1 inflammasomes and signaling pathways involving NF-κB in HaCaT cells. Protein accumulation and mRNA levels were assessed after the application of different amounts of DEP for 24 h. As illustrated in Figure 1A,B, DEP increased the NLRP1 inflammasome, cleaved caspase-1, and cleaved IL-1β, as well as NLRP1 mRNA level. Also, DEP treatment upregulated the NF-κB and IκB phosphorylation (Figure 1C). TXNIP, a piece of the α-arrestin superfamily of proteins, promotes inflammatory responses by binding to inflammasomes in the oxidative stress framework [37]. To elucidate the degree of binding between NLRP1 and TXNIP, western blotting and IP assays were performed, and the binding affinity between the two proteins was strengthened (Figure 1D). These results show that DEP enhances NF-κB signaling and NLRP1 inflammasome expression through the interaction of TXNIP with NLRP1.

### 3.2. IPA Attenuates DEP-Induced NLRP1 Inflammasome Complex in HaCaT Cells

**Figure 2:**
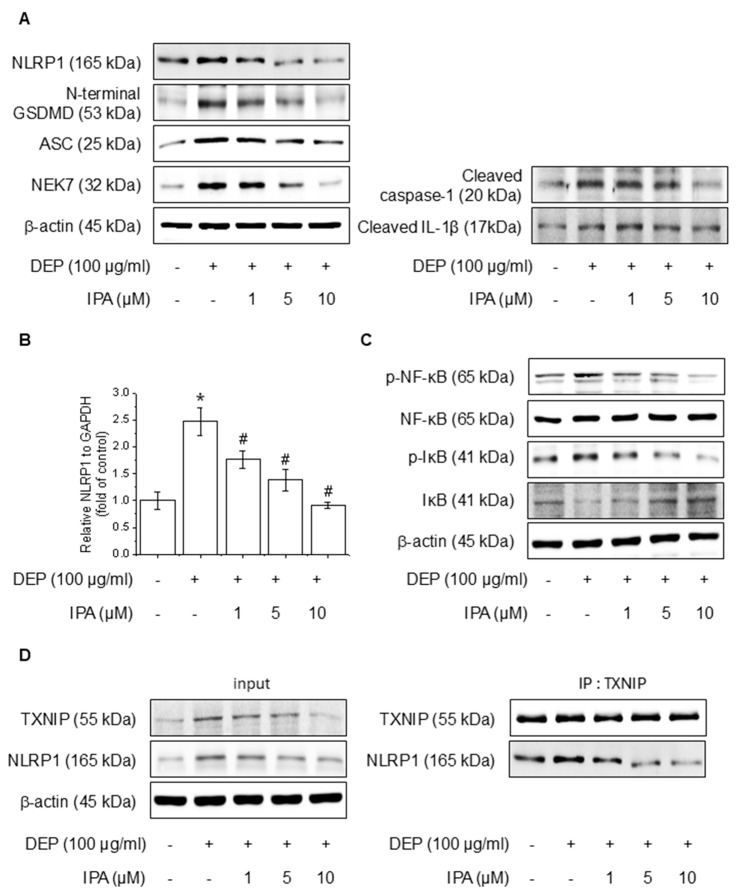
Effect of IPA on the DEP-induced NLRP1 inflammasome complex in HaCaT cells. The cells were treated with 1–10 μM IPA for 12 h before being treated with 100 μg/mL DEP for 24 h. (**A**) The NLRP1 inflammasome complex expression levels were measured by western blot, and (**B**) the NLRP1 mRNA level was confirmed using RT-PCR. Further, cells underwent a pretreatment with 1–10 μM IPA for 12 h, and treatment with 100 μg/mL DEP for 30 min, and the (**C**) NF-κB and IκB phosphorylation levels were represented by western blot. (**D**) After pretreatment of IPA for 12 h, and then DEP for 24 h, the cell lysates were subjected to immunoblotting with indicated antibodies. All experiments were performed thrice (*n* = 3). The data are expressed as the mean ± SD. * *p* < 0.01 compared to the control group. # *p* < 0.01 compared with the DEP treatment group.

We analyzed the effects of IPA on DEP-induced NF-κB signaling and the NLRP1 inflammasome. After pretreatment with 1–10 μM IPA for 12 h, we assessed proteins and mRNA expressions following treatment DEP 100 μg/mL for 24 h. IPA pretreatment decreased the protein of DEP-induced NLRP1 inflammasome, cleaved caspase-1, and IL-1β, and suppressed NLRP1 mRNA levels increased by DEP (Figure 2A,B). Furthermore, IPA significantly attenuated the NF-κB and IκB phosphorylation elevated by DEP (Figure 2C). In addition, IPA reduced the binding of TXNIP to NLRP1, which was enhanced by DEP treatment (Figure 2D), suggesting that IPA exerts anti-inflammatory effects mediated by the modulation of NF-κB signaling and the NLRP1 inflammasome through TXNIP-NLRP1 in HaCaT cells.

### 3.3. IPA Increases Antioxidant Enzyme Gene Expression and Protein Levels

**Figure 3:**
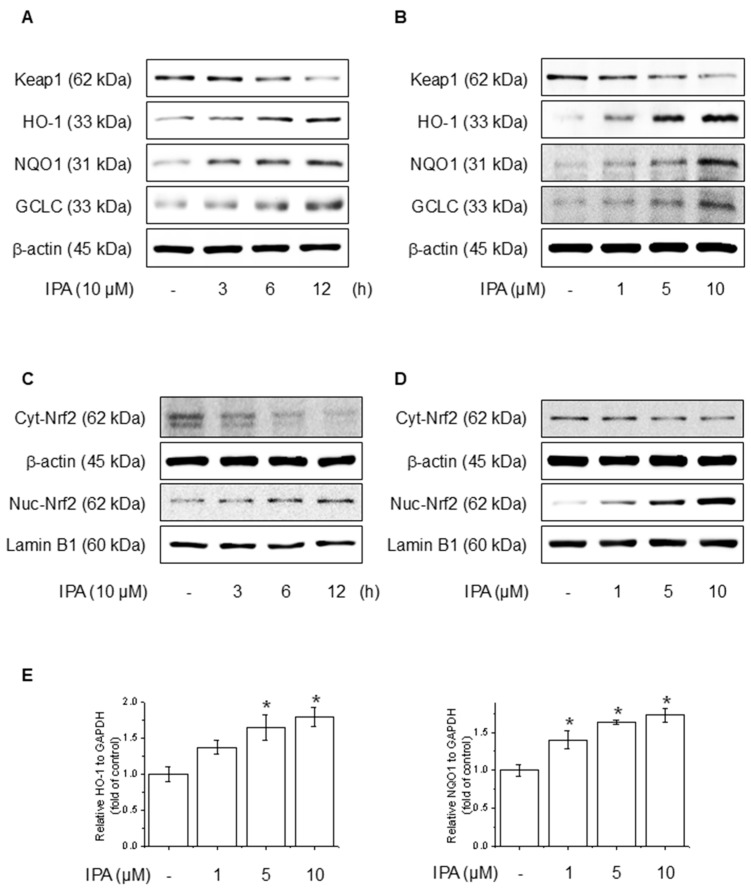
Effect of IPA on antioxidant enzyme by activating the Nrf2-signaling pathway. The cells were treated with 10 μM IPA for 3−12 h and 1−10 μM IPA for 12 h. (**A**,**B**) Keap1 and Nrf2-associated antioxidant enzyme expressions were determined by western blot. (**C**,**D**) Additionally, the nuclear translocation of the Nrf2 was evaluated following treatment with 10 μM IPA for 3–12 h and 1–10 μM IPA for 12 h, with both nuclear and cytosol protein fractions analyzed by western blot. (**E**) The cells were exposed to 1−10 μM for 12 h and HO-1 and NQO1 mRNA levels were assessed through RT-PCR. All experiments were performed thrice (*n* = 3). The data are expressed as the mean ± SD. * *p* < 0.01 compared to the control group.

To evaluate the effect of IPA on the Nrf2 and antioxidant system in HaCaT cells, we conducted experiments at various time points and IPA. HO-1, NQO1, and GCLC concentrations are critical enzymes for cellular antioxidant activity and are regulated by Nrf2, which is modulated by Keap1 [38]. IPA led to the upregulation of HO-1, NQO1, and GCLC, the target genes of Nrf2, in a time- and dose-dependent manner, while simultaneously decreasing Keap1 expression (Figure 3A,B). Moreover, IPA led to a decrease in the cytosolic Nrf2 protein levels while increasing its expression in the nucleus in a time- and concentration-dependent manner (Figure 3C,D). Figure 3E of IPA for 12 h at various concentrations upregulated the mRNA expression of antioxidant enzymes. These data indicated that IPA activated the antioxidant enzyme pathway by increasing Nrf2 transcriptional activity.

### 3.4. IPA Activates Calcium on Nrf2 Expression Through the Akt/AMPK/GSK3β-Signaling Pathway

**Figure 4:**
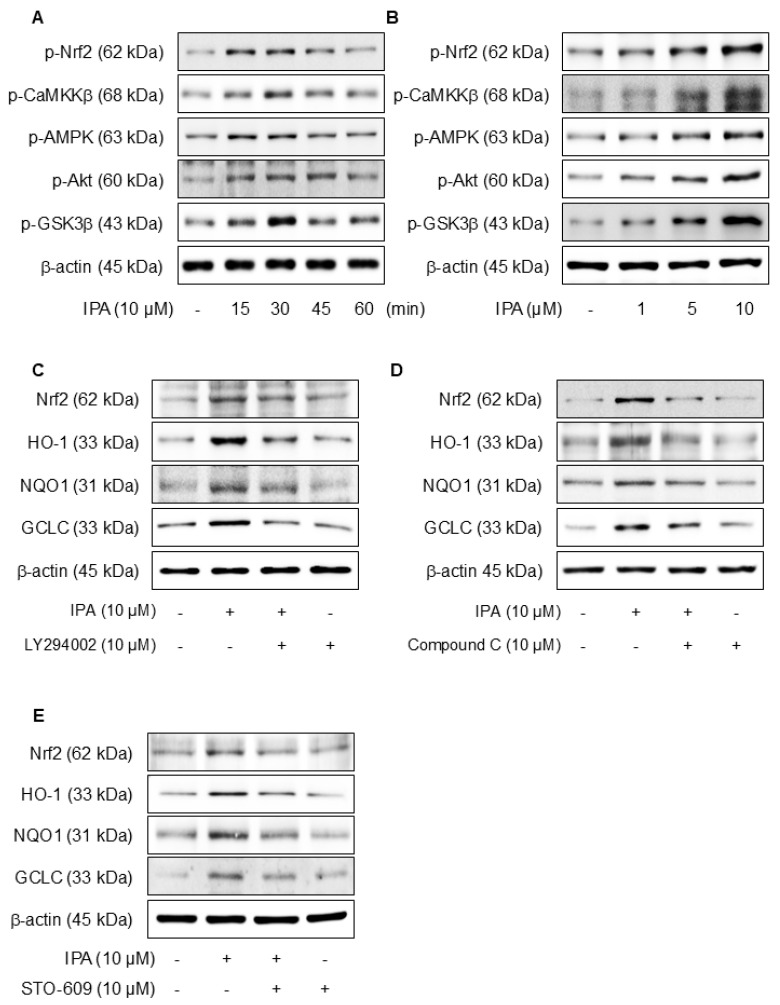

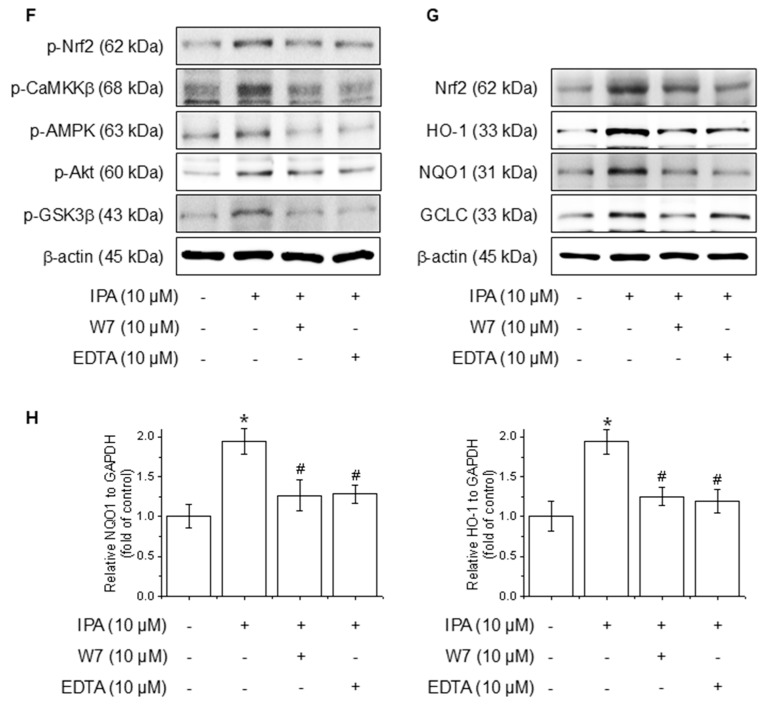
Effect of Ca2+ on IPA-induced Nrf2 expression via the Akt/AMPK/GSK3β-signaling pathway. The cells were treated with 10 μM IPA for 15–60 min and 1–10 μM IPA for 30 min. (**A**,**B**) The CaMKKβ, AMPK, Akt, and GSK3β phosphorylation levels were determined by western blot. In addition, the cells were pretreated with 10 μM LY294002, 10 μM Compound C, and 10 M STO-609 for 1 h, respectively, before treatment with 10 μM IPA for 12 h. (**C**–**E**) Nrf2 and related antioxidant enzyme expression levels were checked by western blot. Effect of Ca^2+^ on IPA-induced Nrf2 expression via the Akt/AMPK/GSK3β-signaling pathway. The cells were subjected to pretreatment with 10 μM W7 and EDTA for 1 h and treated with 10 μM IPA for 30 min. (**F**) The Nrf2, CaMKKβ, AMPK, Akt, and GSK3β phosphorylation levels were measured via western blot. (**G**) The expression levels of Nrf2 and antioxidant enzymes were also determined by western blot. (**H**) The HO-1 and NQO1 mRNA expression levels were assessed using RT-PCR. All experiments were performed thrice (*n* = 3). The data are expressed as the mean ± SD. * *p* < 0.01 compared to the control group. # *p* < 0.01 compared with the IPA treatment group.

We hypothesized that the antioxidant effects of IPA may be due to the activation of Akt, AMPK, and the inhibition of GSK3β. Figure 4A,B show that when IPA was treated at various times and doses, the Nrf2, CaMKKβ, AMPK, Akt, and GSK3β phosphorylation levels all increased. Furthermore, the application of specific inhibitors, LY294002 for Akt, Compound C for AMPK, and STO-609 for CaMKKβ, before IPA treatment, there was a tendency for the increase in Nrf2 and related antioxidant enzymes induced by IPA to diminish (Figure 4C–E). These data infer that IPA facilitates the AMPK and Akt phosphorylation through CaMKKβ and enhances GSK3β-mediated Nrf2 expression.

Calcium ions (Ca^2^⁺) are critical modulators of cellular signaling pathways and contribute to the modulation of inflammatory responses. To determine the influence of calcium-signaling in the generation of Nrf2 expression by IPA over the Akt/AMPK/GSK3β-signaling pathway, the calcium signal was blocked by W7 (calmodulin antagonist) and EDTA (extracellular calcium chelator). The Nrf2, CaMKKβ, AMPK, Akt, and GSK3β phosphorylation increased by IPA were reduced by W7 and EDTA treatment (Figure 4F). W7 and EDTA treatment markedly suppressed both the protein and mRNA levels of Nrf2 and associated antioxidant enzymes stimulated by IPA (Figure 4G,H). These results indicate that IPA modulates Nrf2 expression by activating the calcium-signaling pathways.

### 3.5. IPA Stimulates Nrf2 via an Autophagy-Mediated Pathway

**Figure 5:**
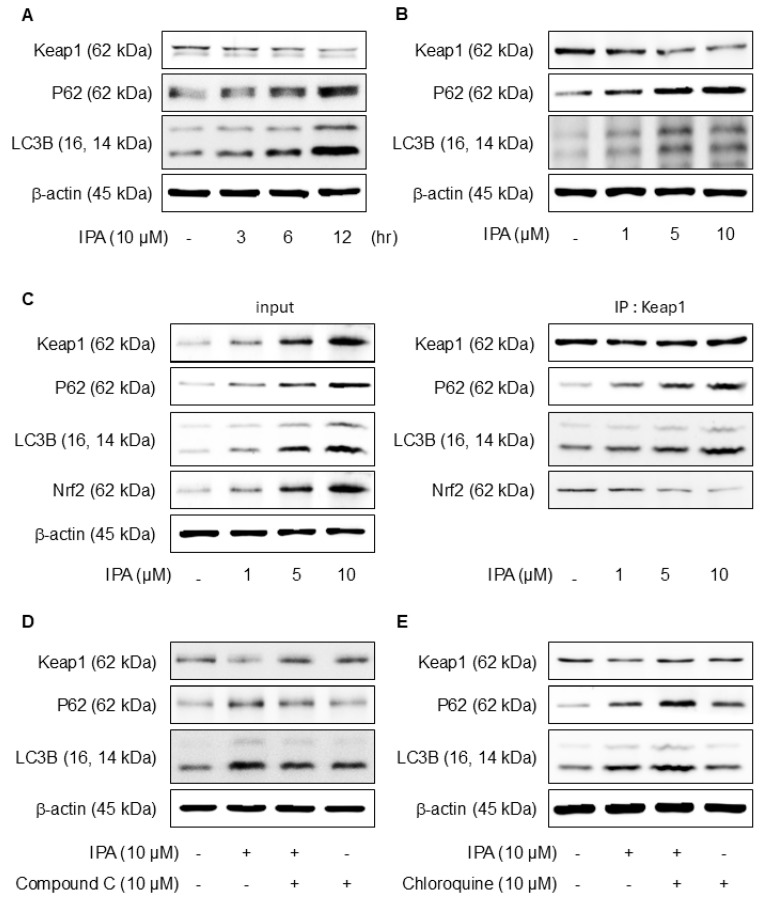
Effect of IPA on Nrf2 activation via the Keap1/p62-signaling pathway. The cells were exposed to 10 μM IPA for 3–12 h and 1–10 μM for 12 h. (**A**,**B**) Keap1, p62, and LC3B expression levels were assayed by western blot. (**C**) The cell lysates were immunoblotted with indicated antibodies following treatment with 1–10 μM IPA for 12 h. After pretreatment of 10 μM Compound C or Chloroquine for 1 h, the cells were treated with 10 μM IPA for 12 h. (**D**,**E**) The Keap1, p62, and LC3B expression levels were measured through western blot. All experiments were performed thrice (*n* = 3). The data are expressed as the mean ± SD.

p62, a gene target of Nrf2, mediates the degradation of Keap-1 [39]. The effects of IPA on autophagy were confirmed experimentally. Treatment with IPA at different time points and concentrations resulted in a decrease in Keap1 expression and an increase in p62 and LC3B expression (Figure 5A,B). IP assays were conducted to elucidate the interactions between Keap1, p62, and LC3B, revealing strong binding of Nrf2, p62, and LC3B to Keap1 (Figure 5C). When IPA was administered after the administration of Compound C, an AMPK inhibitor, IPA-reduced Keap1 increased, and the expression of IPA-induced p62 and LC3B decreased upon treatment with the inhibitor (Figure 5D). Moreover, the autophagy inhibitor chloroquine increased Keap1, which was reduced by IPA, and further elevated the p62 and LC3B protein expression, which was increased by IPA (Figure 5E). These results suggest that IPA influences autophagy flux.

### 3.6. IPA Suppresses ROS Production on DEP-Induced NLRP1 Inflammasome Through the Nrf2-Signaling Pathway

**Figure 6:**
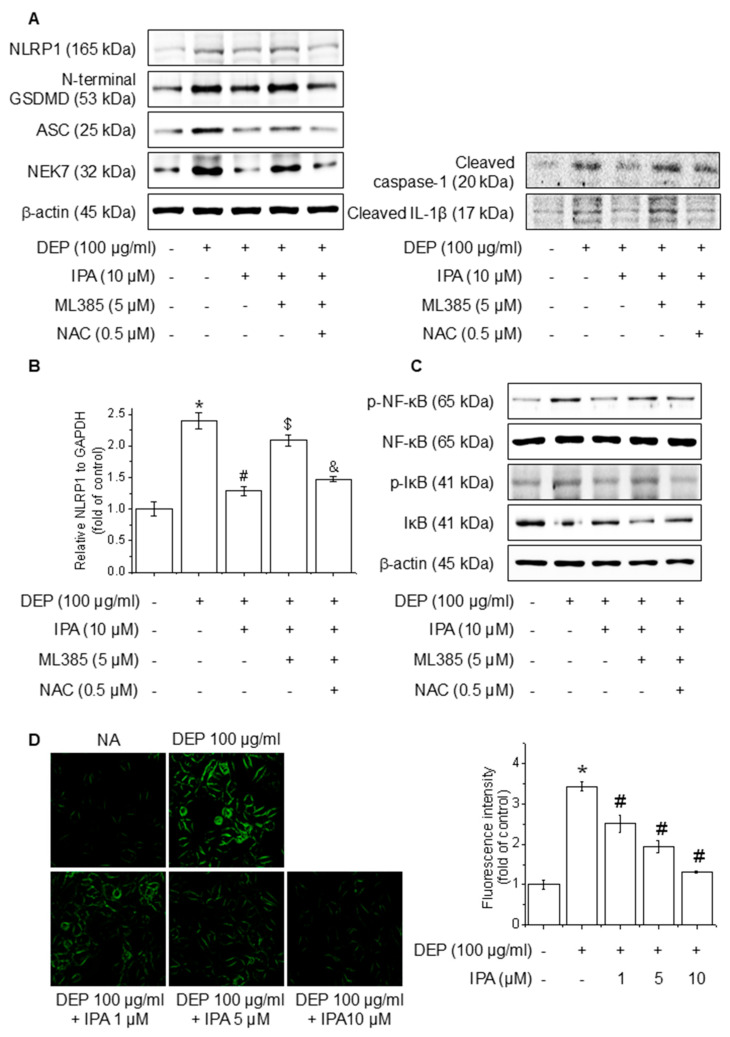
Effect of ROS generation on DEP-induced NLRP1 inflammasome through the Nrf2-signaling pathway. The cells were cultured with 0.5 μM NAC for 1 h before adding 5 μM ML385 for 1 h before the addition of 10 μM IPA for 12 h, followed by 100 μg/mL DEP for 24 h. (**A**) The expression levels of NLRP1 inflammasome were performed with western blot. (**B**) NLRP1 mRNA expression level was evaluated by RT-PCR. The cells were maintained with 0.5 μM NAC for 1 h before the addition of 5 μM ML385 for 1 h, followed by 10 μM IPA for 12 h, and then 100 μg/mL DEP for 30 min. (**C**) NF-κB and IκB phosphorylation levels were detected by western blot. (**D**) ROS accumulation was detected employing H2DCFDA and the quantification of ROS reduction, as indicated. All experiments were performed thrice (*n* = 3). The data are expressed as the mean ± SD. * *p* < 0.01 compared to the control group. # *p* < 0.01 compared with DEP treatment group. $ *p* < 0.01 compared with the DEP and IPA treatment groups. and & *p* < 0.01 compared with the DEP, IPA, and ML385 treatment groups.

To investigate the function of ROS mediated via the Nrf2 pathway in the IPA inhibition of the DEP-induced NLRP1 inflammasome, we pretreated cells with the ROS inhibitor NAC in the presence of ML385 and IPA. The treatment of NAC abolished the reversal effect of ML385 on the NLRP1 inflammasome, cleaved caspase-1, cleaved IL-1β, and NLRP1 mRNA level inhibited by IPA, leading to a reduction in their expression (Figure 6A,B). Furthermore, NAC treatment invalidated the impact of ML385 on the NF-κB and IκB phosphorylation suppressed by IPA (Figure 6C). The ROS generated by DEP was diminished by 62% following pretreatment with IPA (Figure 6D). This suggests that IPA suppresses NF-κB-signaling and NLRP1 inflammasome activation by suppressing ROS generation via the Nrf2-signaling pathway.

## 4. Discussion

Inflammation is a crucial immune response that protects the body from damage and infections. However, when in excess, it can precipitate various diseases. Upon the recognition of harmful stimuli, inflammatory cytokines and related inflammatory signaling pathways are activated [11,12]. IPA, isolated from *A. Koreanum*, is a lupane-type triterpenoid that protects against vascular endothelial dysfunction and inflammatory diseases [32,33]. This study revealed that IPA inhibited DEP-induced NLRP1 inflammasome activation via the Keap1/p62/Nrf2-signaling pathway in HaCaT keratinocytes. Exposure to PM2.5 induces oxidative stress, and the role of the NLRP3 inflammasome has been extensively investigated. Previous studies have shown that exposure to PM2.5 induces oxidative stress and activates the NLRP1 inflammasome, contributing to skin inflammation and damage [9]; however, this has not been widely studied. Additionally, an association between ROS and inflammasome activation has been reported [40]. Our results corroborate those of previous studies by demonstrating that DEP triggers the activation of the NLRP1 inflammasome and NF-κB signaling through ROS generation. Additionally, TXNIP, which plays an important role in oxidative stress-dependent inflammatory signaling, promotes inflammation by increasing ROS levels and interacting with inflammasomes [37]. In the current study, we determined that the DEP process leads to TXNIP expression enhancement, which is associated with NLRP1. The regulation of antioxidant enzyme expression by Nrf2 is intricately linked to its anti-inflammatory effects, particularly in diminishing oxidative stress, specifically ROS [41]. Consequently, this study demonstrates that IPA inhibited not only ROS but also NLRP1 inflammasome and the NF-κB-signaling pathways. Our study emphasizes the role of IPA in mediating these inhibitory effects and provides new insights into its antioxidant and anti-inflammatory properties.

Nrf2 is recognized for its protective function in mitigating inflammation through the modulation of the expression of antioxidant enzymes, including HO-1, NQO1, and GCLC. HO-1 exerts anti-inflammatory effects by inhibiting NF-κB signaling through carbon monoxide formation [42]. Keap1, which is degraded by the ubiquitin-proteasome framework, interacts with Nrf2, thereby reducing its stability and preventing its function as a transcription factor [38]. In this study, IPA increased the Nrf2, HO-1, NQO1, and GCLC protein levels, promoted the nuclear translocation of Nrf2, and significantly suppressed Keap1 expression. Moreover, Nrf2 inhibition reversed the suppressive effects of IPA on the DEP-induced NLRP1 inflammasome and the NF-κB-signaling pathways. Therefore, we propose that IPA exerts its protective effects against inflammation by regulating antioxidant enzymes via the Nrf2-signaling pathway. When Nrf2 is phosphorylated, Nrf2 exhibits enhanced stability and promotes its translocation to the nucleus, thereby upregulating the expression of antioxidant genes.

AMPK, an important cell metabolism regulator, intervenes in Nrf2 activation to activate antioxidant enzyme expression while suppressing the inflammatory response [43]. Additionally, AMPK stimulates the PI3K/Akt-signaling pathway activation [44]. Akt regulates cell proliferation and survival by modulating various intracellular signals [45]. AMPK activates Akt, which induces GSK3β phosphorylation at serine 9, promoting the nuclear translocation and transcriptional upregulation of Nrf2 by stabilizing it [44]. CaMKKβ, an upstream AMPK regulator, is recognized for its involvement in AMPK phosphorylation in response to intracellular calcium fluctuations [46]. Calcium, a vital component of cellular signaling pathways, regulates inflammatory responses and promotes the expression of antioxidant enzymes. Our findings indicated that IPA enhances the CaMKKβ and AMPK, Akt, and GSK3β, while the inhibition of Akt, AMPK, and CaMKKβ phosphorylation attenuates the IPA-induced Nrf2 and associated antioxidant enzymes. Furthermore, blocking calcium signaling inhibited the IPA-triggered CaMKKβ, AMPK, Akt, and GSK3β phosphorylation, as well as the expression of antioxidant enzymes. Thus, we propose that IPA activates CaMKKβ through calcium signaling, leading to Nrf2 expression through the AMPK/Akt/GSK3β-signaling pathway, exerting anti-inflammatory effects.

Autophagy is a critical cellular mechanism that eliminates abnormal components and recycles them for survival and function. P62/SQSTM1 is an adaptor protein integral to the autophagy process and is recognized as a target gene of Nrf2; it binds to Keap1, stabilizes Nrf2, and activates the Nrf2-Keap1 pathway [47]. P62 has been reported to interact with LC3B, a protein crucial for autophagosome development and maturation [47]. Additionally, p62 is phosphorylated and accumulated by AMPK, which promotes the autophagy process [48].

The results demonstrated that IPA induces p62 and LC3B expression, facilitating the separation of Nrf2 from Keap1. Importantly, the IPA-induced expression of p62 and LC3B was suppressed upon treatment with an AMPK inhibitor. Furthermore, the inhibition of autophagy resulted in the accumulation of IPA-induced p62 and further LC3B expression elevation. Therefore, IPA activates Nrf2 by promoting the degradation of Keap1 through p62 accumulation via autophagosome formation.

PM2.5 is known to activate multiple inflammasomes, including NLRP3 and NLRP1 [5]. In this study, we focused on the NLRP1 inflammasome pathway, which is predominantly expressed and functionally significant in keratinocytes. Unlike NLRP3, which is primarily activated in immune cells such as macrophages and dendritic cells, NLRP1 plays a more prominent role in epithelial tissues, particularly the skin, in response to environmental stressors such as UV radiation and particulate matter [23,49]. Given that HaCaT cells are a keratinocyte model, targeting NLRP1 provides a physiologically relevant approach to understanding DEP-induced cutaneous inflammasome activation.

Our findings are based on experiments conducted using HaCaT keratinocyte cell line, which, while widely used and convenient for in vitro studies, does not fully recapitulate the behavior of primary human keratinocytes, particularly in the context of innate immune signaling. However, HaCaT cells remain a well-established and reproducible in vitro model for studying keratinocyte biology and skin-related mechanisms. Importantly, they retain key functional characteristics relevant to our study context such as epidermal structure, some immune response pathways and response to stress or drug. To enhance the physiological relevance of our results, future studies will include validation using primary human keratinocytes or 3D reconstructed epidermis models that more closely mimic the in vivo skin environment. Moreover, we observed that IPA demonstrated anti-inflammatory activity at concentrations between 1 and 10 µM in vitro. However, the specific data on the maximum non-toxic concentration of IPA in keratinocytes beyond 10 µM is currently limited. Give the structural similarity of IPA to other triterpenoids such as 18β-glycyrrhetinic acid which has shown cytotoxic effects in HaCaT cells at concentrations above 50 µM [50], it is plausible that IPA may exhibit a comparable cytotoxicity profile, indicating a promising therapeutic window. While dermal pharmacokinetics of IPA remain to be characterized, similar triterpenoids have been proved to reach effective epidermal levels through optimized topical formulations such as liposomes, nanoemulsions, and solid lipid nanoparticles [51,52,53]. These systems can enhance dermal delivery and retention, potentially enabling IPA to achieve therapeutic concentrations in the viable layers of the skin. Additional research, including skin permeation and pharmacokinetic studies, will be needed to determine whether therapeutically relevant concentrations of IPA can be achieved in vivo.

In summary, this study elucidates that PM2.5 stimulates the NLRP1 inflammasome and NF-κB-signaling pathways through ROS generation. Additionally, IPA induces the CaMKKβ, AMPK, Akt, and GSK3β phosphorylation according to calcium influx, inhibiting the NLRP1 inflammasome through the upregulation of Nrf2 and antioxidant enzymes.

Finally, we confirmed that IPA exerted anti-inflammatory effects by diminishing inflammasomes in DEP-exposed keratinocytes. The anti-inflammatory effects of IPA are driven by its antioxidant enzyme activity via the Keap1/p62/Nrf2 pathway. Overall, we propose that IPA is a potential therapeutic candidate for addressing skin inflammation by modulating the inflammatory response.

## 5. Conclusions

In this study, we revealed that IPA inhibits the DEP-induced NF-κB-signaling pathway and NLRP1 inflammasome. IPA also reduced ROS production through the Keap-1/p62/Nrf2-signaling pathway. Collectively, these results imply that IPA can suppress the inflammation caused by PM2.5 in human keratinocytes by augmenting antioxidant enzyme expression.

## Data Availability

Data will be made available on request.

## Supplementary Materials

The following supporting information can be downloaded at: https://www.mdpi.com/article/10.3390/antiox14050610/s1. Figure S1: Effect of IPA on DEP-induced cytotoxicity in HaCaT cells, Figure S2: Effect of DEP on NLRP1 inflammasome complex in HaCaT cells, Figure S3: Effect of IPA on the DEP-induced NLRP1 inflammasome complex in HaCaT cells, Figure S4: Effect of IPA on antioxidant enzyme by activating Nrf2 signaling pathway, Figure S5: Effect of Ca^2+^ on IPA-induced Nrf2 expression via the Akt/AMPK/GSK3β signaling pathway, Figure S6: Effect of IPA on Nrf2 activation via the Keap1/p62 signaling pathway, Figure S7: Effect of ROS generation on DEP-induced NLRP1 inflammasome through the Nrf2 signaling pathway.

## Abbreviations

The following abbreviations are used in this manuscript:

CARD	C-terminal caspase activation and recruitment domain
DEP	Diesel exhaust particulate matter
DMSO	Dimethylsulfoxide
GSDMD	Gasdermin D
IL	Interleukin
IPA	Impressic Acid
NLRP1	NOD-like receptor protein1
PM2.5	Particulate matter
PYD	Pyrin domain
ROS	Reactive oxygen species
TNF-α	Tumor Necrosis Factor-alpha

## References

[1] Farahani V.J., Pirhadi M., Sioutas C. (2021). Are standardized diesel exhaust particles (DEP) representative of ambient particles in air pollution toxicological studies?. Sci. Total Environ..

[2] Dai P., Shen D., Shen J., Tang Q., Xi M., Li Y., Li C. (2019). The roles of Nrf2 and autophagy in modulating inflammation mediated by TLR4-NFκB in A549 cell exposed to layer house particulate matter 2.5 (PM_2.5_). Chemosphere.

[3] Paarwater B.A., Mouton J.M., Sampson S.L., Malherbe S.T., Shaw J.A., Walzl G., Kotze L.A., du Plessis N. (2021). Inhaled particulate matter affects immune responsiveness of human lung phagocytes to mycobacteria. Am. J. Physiol. Lung Cell Mol. Physiol.

[4] Badran G., Verdin A., Grare C., Abbas I., Achour D., Ledoux F., Roumie M., Cazier F., Courcot D., Lo Guidice J.M. (2020). Toxicological appraisal of the chemical fractions of ambient fine (PM_2.5-0.3_) and quasi-ultrafine (PM_0.3_) particles in human bronchial epithelial BEAS-2B cells. Environ. Pollut..

[5] Dong L., Hu R., Yang D., Zhao J., Kan H., Tan J., Guan M., Kang Z., Xu F. (2020). Fine Particulate Matter (PM_2.5_) upregulates expression of Inflammasome NLRP1 via ROS/NF-κB signaling in HaCaT Cells. Int. J. Med. Sci..

[6] Moon J.Y., Ngoc L.T.N., Chae M., Tran V.V., Lee Y.C. (2020). Effects of Microwave-Assisted *Opuntia humifusa* Extract in Inhibiting the Impacts of Particulate Matter on Human Keratinocyte Skin Cell. Antioxidants.

[7] Henrick B.M., Rodriguez L., Lakshmikanth T., Pou C., Henckel E., Arzoomand A., Olin A., Wang J., Mikes J., Tan Z. (2021). Bifidobacteria-mediated immune system imprinting early in life. Cell.

[8] Ahn E.K., Yoon H.K., Jee B.K., Ko H.J., Lee K.H., Kim H.J., Lim Y. (2008). COX-2 expression and inflammatory effects by diesel exhaust particles in vitro and in vivo. Toxicol. Lett..

[9] Chen R., Li H., Cai J., Wang C., Lin Z., Liu C., Niu Y., Zhao Z., Li W., Kan H. (2018). Fine Particulate Air Pollution and the Expression of microRNAs and Circulating Cytokines Relevant to Inflammation, Coagulation, and Vasoconstriction. Environ. Health Perspect..

[10] Li H., Cai J., Chen R., Zhao Z., Ying Z., Wang L., Chen J., Hao K., Kinney P.L., Chen H. (2017). Particulate Matter Exposure and Stress Hormone Levels: A Randomized, Double-Blind, Crossover Trial of Air Purification. Circulation.

[11] Niebler M., Qian X., Höfler D., Kogosov V., Kaewprag J., Kaufmann A.M., Ly R., Böhmer G., Zawatzky R., Rösl F. (2013). Post-translational control of IL-1β via the human papillomavirus type 16 E6 oncoprotein: A novel mechanism of innate immune escape mediated by the E3-ubiquitin ligase E6-AP and p53. PLoS Pathog..

[12] Zhou J.Y., Sarkar M.K., Okamura K., Harris J.E., Gudjonsson J.E., Fitzgerald K.A. (2023). Activation of the NLRP1 inflammasome in human keratinocytes by the dsDNA mimetic poly (dA:dT). Porc. Natl. Acad. Sci. USA.

[13] Guo H., Callaway J.B., Ting J.P. (2015). Inflammasomes: Mechanism of action, role in disease, and therapeutics. Nat. Med..

[14] Beer H.D., Contassot E., French L.E. (2014). The inflammasomes in autoinflammatory diseases with skin involvement. J. Investig. Dermatol..

[15] Zheng D., Liwinski T., Elinav E. (2020). Inflammasome activation and regulation: Toward a better understanding of complex mechanisms. Cell Discov.

[16] Faustin B., Lartigue L., Bruey J.M., Luciano F., Sergienko E., Bailly-Maitre B., Volkmann N., Hanein D., Rouiller I., Reed J.C. (2007). Reconstituted NALP1 inflammasome reveals two-step mechanism of caspase-1 activation. Mol. Cell.

[17] Burian M., Yazdi A.S. (2018). NLRP1 Is the Key Inflammasome in Primary Human Keratinocytes. J. Investig. Dermatol..

[18] Franklin B.S., Latz E., Schmidt F.I. (2018). The intra- and extracellular functions of ASC specks. Immunol. Rev..

[19] Fairbrother W.J., Gordon N.C., Humke E.W., O’Rourke K.M., Starovasnik M.A., Yin J.P., Dixit V.M. (2001). The PYRIN domain: A member of the death domain-fold superfamily. Protein Sci..

[20] Mantovani A., Allavena P., Sica A., Balkwill F. (2008). Cancer-related inflammation. Nature.

[21] Rathinam V.A.K., Fitzgerald K.A. (2016). Inflammasome Complexes: Emerging Mechanisms and Effector Functions. Cell.

[22] Kaufmann S.H., Hengartner M.O. (2001). Programmed cell death: Alive and well in the new millennium. Trends Cell Biol..

[23] Fenini G., Karakaya T., Hennig P., Filippo M.D., Beer H.D. (2020). The NLRP1 Inflammasome in Human Skin and Beyond. Int. J. Mol. Sci..

[24] Ciążyńska M., Bednarski I.A., Wódz K., Narbutt J., Lesiak A. (2020). NLRP1 and NLRP3 inflammasomes as a new approach to skin carcinogenesis. Oncol. Lett..

[25] Keller M., Rüegg A., Werner S., Beer H.D. (2008). Active caspase-1 is a regulator of unconventional protein secretion. Cell.

[26] Aglietti R.A., Dueber E.C. (2017). Recent Insights into the Molecular Mechanisms Underlying Pyroptosis and Gasdermin Family Functions. Trends Immunol..

[27] Dombrowski Y., Peric M., Koglin S., Kammerbauer C., Göss C., Anz D., Simanski M., Gläser R., Harder J., Hornung V. (2011). Cytosolic DNA triggers inflammasome activation in keratinocytes in psoriatic lesions. Sci. Transl. Med..

[28] Liu X., Zhang Z., Ruan J., Pan Y., Magupalli V.G., Wu H., Lieberman J. (2016). Inflammasome-activated gasdermin D causes pyroptosis by forming membrane pores. Nature.

[29] Dinarello C.A. (2009). Immunological and inflammatory functions of the interleukin-1 family. Annu. Rev. Immunol..

[30] Han E.J., Fernando I.P.S., Kim H.S., Lee D.S., Kim A., Je J.G., Seo M.J., Jee Y.H., Jeon Y.J., Kim S.Y. (2021). (-)-Loliolide Isolated from *Sargassum horneri* Suppressed Oxidative Stress and Inflammation by Activating Nrf2/HO-1 Signaling in IFN-γ/TNF-α-Stimulated HaCaT Keratinocytes. Antioxidants.

[31] Kim J.A., Yang S.Y., Koo J.E., Koh Y.S., Kim Y.H. (2010). Lupane-type triterpenoids from the steamed leaves of Acanthopanax koreanum and their inhibitory effects on the LPS-stimulated pro-inflammatory cytokine production in bone marrow-derived dendritic cells. Bioorg. Med. Chem. Lett..

[32] Choi J.H., Lee G.H., Jin S.W., Kim J.Y., Hwang Y.P., Han E.H., Kim Y.H., Jeong H.G. (2021). Impressic Acid Ameliorates Atopic Dermatitis-Like Skin Lesions by Inhibiting ERK1/2-Mediated Phosphorylation of NF-κB and STAT1. Int. J. Mol. Sci..

[33] Lee G.H., Kim J.Y., Jin S.W., Pham T.H., Park J.S., Kim C.Y., Choi J.H., Han E.H., Kim Y.H., Jeong H.G. (2021). Impressic Acid Attenuates the Lipopolysaccharide-Induced Inflammatory Response by Activating the AMPK/GSK3β/Nrf2 Axis in RAW264.7 Macrophages. Int. J. Mol. Sci..

[34] Lim H., Min D.S., Yun H.E., Kim K.T., Sun Y.N., Dat L.D., Kim Y.H., Kim H.P. (2017). Impressic acid from Acanthopanax koreanum, possesses matrix metalloproteinase-13 down-regulating capacity and protects cartilage destruction. J. Ethnopharmacol..

[35] Kim J.A., Yang S.Y., Song S.B., Kim Y.H. (2011). Effects of impressic acid from Acanthopanax koreanum on NF-κB and PPARγ activities. Arch. Pharm. Res..

[36] National Institute of Standards and Technology (NIST) (2021). Standard Reference Material 2975: Diesel Particulate Matter (Industrial Forklift).

[37] Ye X., Zuo D., Yu L., Zhang L., Tang J., Cui C., Bao L., Zan K., Zhang Z., Yang X. (2017). ROS/TXNIP pathway contributes to thrombin induced NLRP3 inflammasome activation and cell apoptosis in microglia. Biochem. Biophys. Res. Commun..

[38] Shi L., Hao Z., Zhang S., Wei M., Lu B., Wang Z., Ji L. (2018). Baicalein and baicalin alleviate acetaminophen-induced liver injury by activating Nrf2 antioxidative pathway: The involvement of ERK1/2 and PKC. Biochem. Pharmacol..

[39] Nishikawa S., Inoue Y., Hori Y., Miyajima C., Morishita D., Ohoka N., Hida S., Makino T., Hayashi H. (2020). Anti-Inflammatory Activity of Kurarinone Involves Induction of HO-1 via the KEAP1/Nrf2 Pathway. Antioxidants.

[40] Mittal M., Siddiqui M.R., Tran K., Reddy S.P., Malik A.B. (2014). Reactive oxygen species in inflammation and tissue injury. Antioxid. Redox Signal..

[41] Zhang H.F., Wang J.H., Wang Y.L., Gao C., Gu Y.T., Huang J., Wang J.H., Zhang Z. (2019). Salvianolic Acid A Protects the Kidney against Oxidative Stress by Activating the Akt/GSK-3β/Nrf2 Signaling Pathway and Inhibiting the NF-κB Signaling Pathway in 5/6 Nephrectomized Rats. Oxid. Med. Cell. Longev..

[42] Ren J., Su D., Li L., Cai H., Zhang M., Zhai J., Li M., Wu X., Hu K. (2020). Anti-inflammatory effects of Aureusidin in LPS-stimulated RAW264.7 macrophages via suppressing NF-κB and activating ROS- and MAPKs-dependent Nrf2/HO-1 signaling pathways. Toxicol. Appl. Pharmacol..

[43] Mo C., Wang L., Zhang J., Numazawa S., Tang H., Tang X., Han X., Li J., Yang M., Wang Z. (2014). The crosstalk between Nrf2 and AMPK signal pathways is important for the anti-inflammatory effect of berberine in LPS-stimulated macrophages and endotoxin-shocked mice. Antioxid. Redox. Signal..

[44] Wang L., Zhang S., Cheng H., Lv H., Cheng G., Ci X. (2016). Nrf2-mediated liver protection by esculentoside A against acetaminophen toxicity through the AMPK/Akt/GSK3β pathway. Free Radic. Biol. Med..

[45] Xu N., Lao Y., Zhang Y., Gillespie D.A. (2012). Akt: A double-edged sword in cell proliferation and genome stability. J. Oncol..

[46] Nakanishi A., Hatano N., Fujiwara Y., Sha’ri A., Takabatake S., Akano H., Kanayama N., Magari M., Nozaki N., Tokumitsu H. (2017). AMP-activated protein kinase-mediated feedback phosphorylation controls the Ca2+/calmodulin (CaM) dependence of Ca2+/CaM-dependent protein kinase kinase β. J. Biol. Chem..

[47] Wei R., Enaka M., Muragaki Y. (2019). Activation of KEAP1/NRF2/P62 signaling alleviates high phosphate-induced calcification of vascular smooth muscle cells by suppressing reactive oxygen species production. Sci. Rep..

[48] Ha S., Jeong S.H., Yi K., Chung K.M., Hong C.J., Kim S.W., Kim E.K., Yu S.W. (2017). Phosphorylation of p62 by AMP-activated protein kinase mediates autophagic cell death in adult hippocampal neural stem cells. J. Biol. Chem.

[49] Calabrese L., Fiocco Z., Mellett M., Aoki R., Rubegni P., French L.E., Satoh T.K. (2024). Role of the NLRP1 inflammasome in skin cancer and inflammatory skin diseases. Br. J. Dermatol..

[50] Gao J., Guo J., Nong Y., Mo W., Fang H., Mi J., Qi Q., Yang M. (2020). 18β-Glycyrrhetinic acid induces human HaCaT keratinocytes apoptosis through ROS-mediated PI3K-Akt signaling pathway and ameliorates IMQ-induced psoriasis-like skin lesions in mice. BMC Pharmacol. Toxicol..

[51] Milan A., Mioc A., Prodea A., Mioc M., Buzatu R., Ghiulai R., Racoviceanu R., Caruntu F., Socia C. (2022). The Optimized Delivery of Triterpenes by Liposomal Nanoformulations: Overcoming the Challenges. Int. J. Mol. Sci..

[52] Stefanov S.R., Andonova V.Y. (2021). Lipid Nanoparticulate Drug Delivery Systems: Recent Advances in the Treatment of Skin Disorders. Pharmaceuticals.

[53] Liu M., Wen J., Sharma M. (2020). Solid Lipid Nanoparticles for Topical Drug Delivery: Mechanisms, Dosage Form Perspectives, and Translational Status. Curr. Pharm. Des..

