# Supplementary material for: Anti-Inflammasome Effect of Impressic Acid on Diesel Exhaust Particulate Matter-Induced NLRP1 Inflammasome via the Keap1/p62/Nrf2-Signaling Pathway in Keratinocytes
Source: Antioxidants (Basel). 2025 May 19;14(5):610. doi: 10.3390/antiox14050610 (PMC12109400; doi:10.3390/antiox14050610)
Supplement: Supplementary file 1 [file antioxidants-14-00610-s001.zip › antioxidants-3608288-supplementary.pptx]

## Slide 1
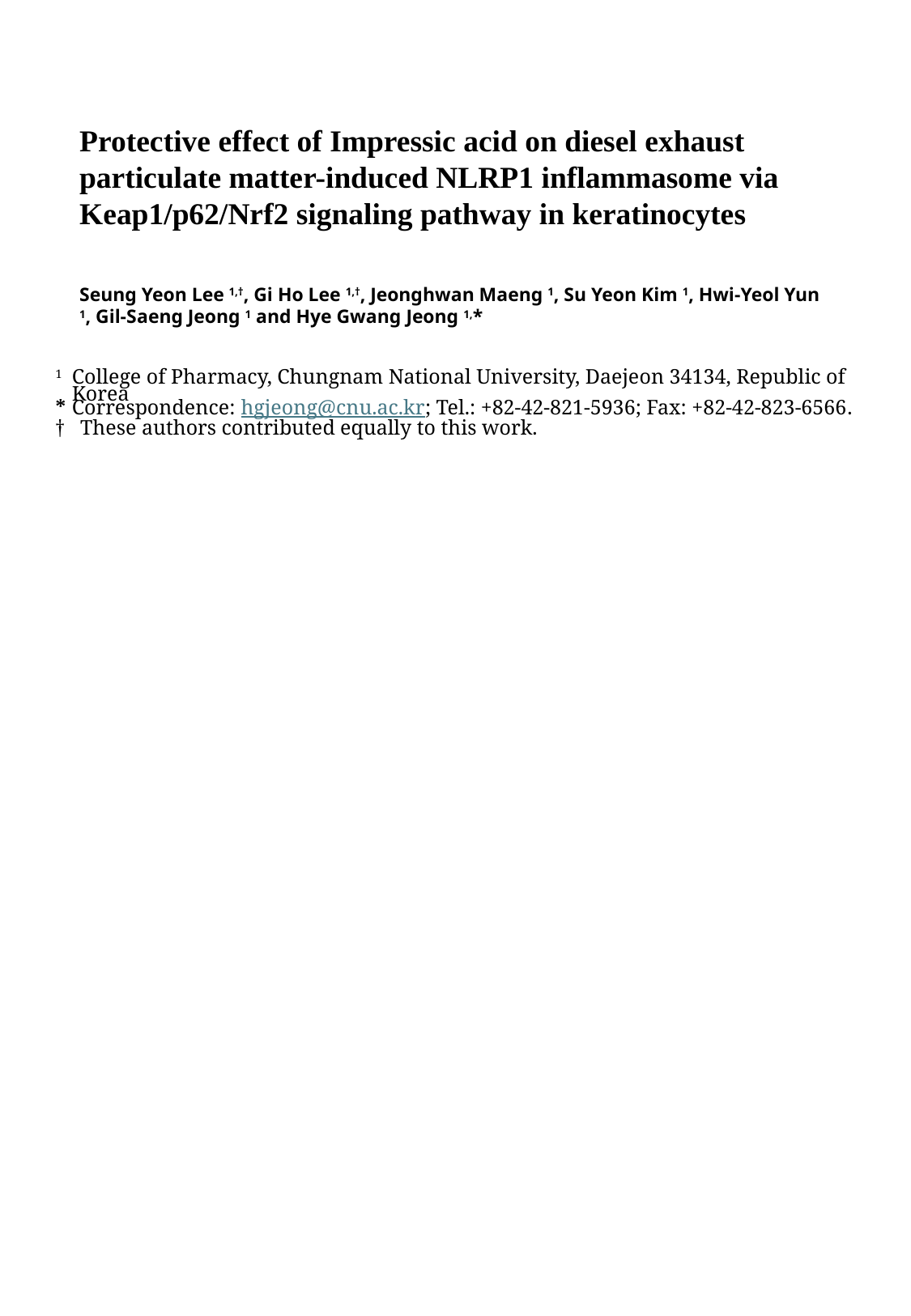

Protective effect of Impressic acid on diesel exhaust particulate matter-induced NLRP1 inflammasome via Keap1/p62/Nrf2 signaling pathway in keratinocytes
Seung Yeon Lee 1,†, Gi Ho Lee 1,†, Jeonghwan Maeng 1, Su Yeon Kim 1, Hwi-Yeol Yun 1, Gil-Saeng Jeong 1 and Hye Gwang Jeong 1,*
1	College of Pharmacy, Chungnam National University, Daejeon 34134, Republic of Korea
*	Correspondence: hgjeong@cnu.ac.kr; Tel.: +82-42-821-5936; Fax: +82-42-823-6566.
† These authors contributed equally to this work.

## Slide 2
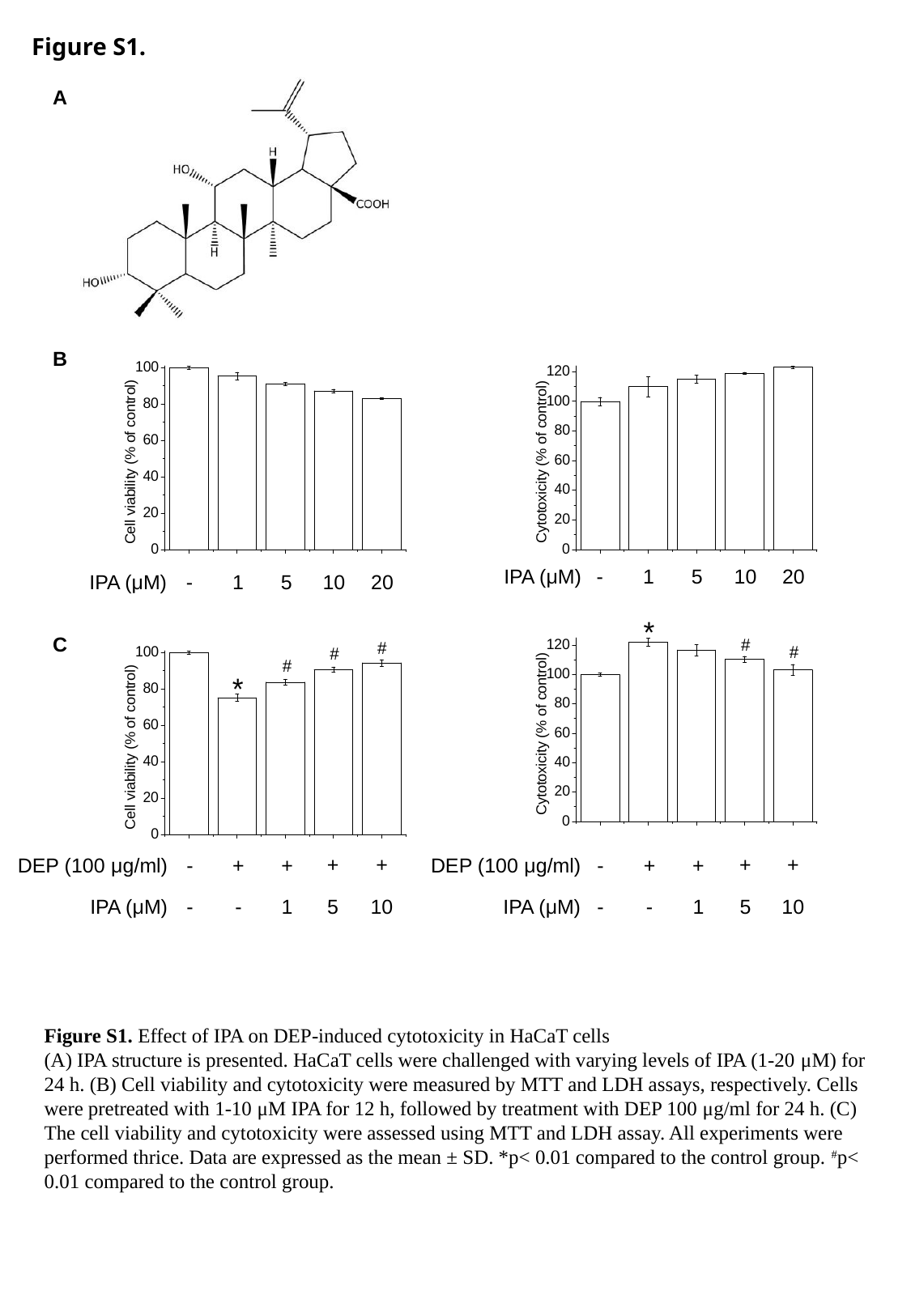

Figure S1.
A
IPA (μM)
-
1
5
10
20
IPA (μM)
-
1
5
10
20
B
+
+
DEP (100 μg/ml)
-
+
+
IPA (μM)
-
-
1
5
10
+
+
DEP (100 μg/ml)
-
+
+
IPA (μM)
-
-
1
5
10
C
Figure S1. Effect of IPA on DEP-induced cytotoxicity in HaCaT cells
(A) IPA structure is presented. HaCaT cells were challenged with varying levels of IPA (1-20 μM) for 24 h. (B) Cell viability and cytotoxicity were measured by MTT and LDH assays, respectively. Cells were pretreated with 1-10 μM IPA for 12 h, followed by treatment with DEP 100 μg/ml for 24 h. (C) The cell viability and cytotoxicity were assessed using MTT and LDH assay. All experiments were performed thrice. Data are expressed as the mean ± SD. *p< 0.01 compared to the control group. #p< 0.01 compared to the control group.

## Slide 3
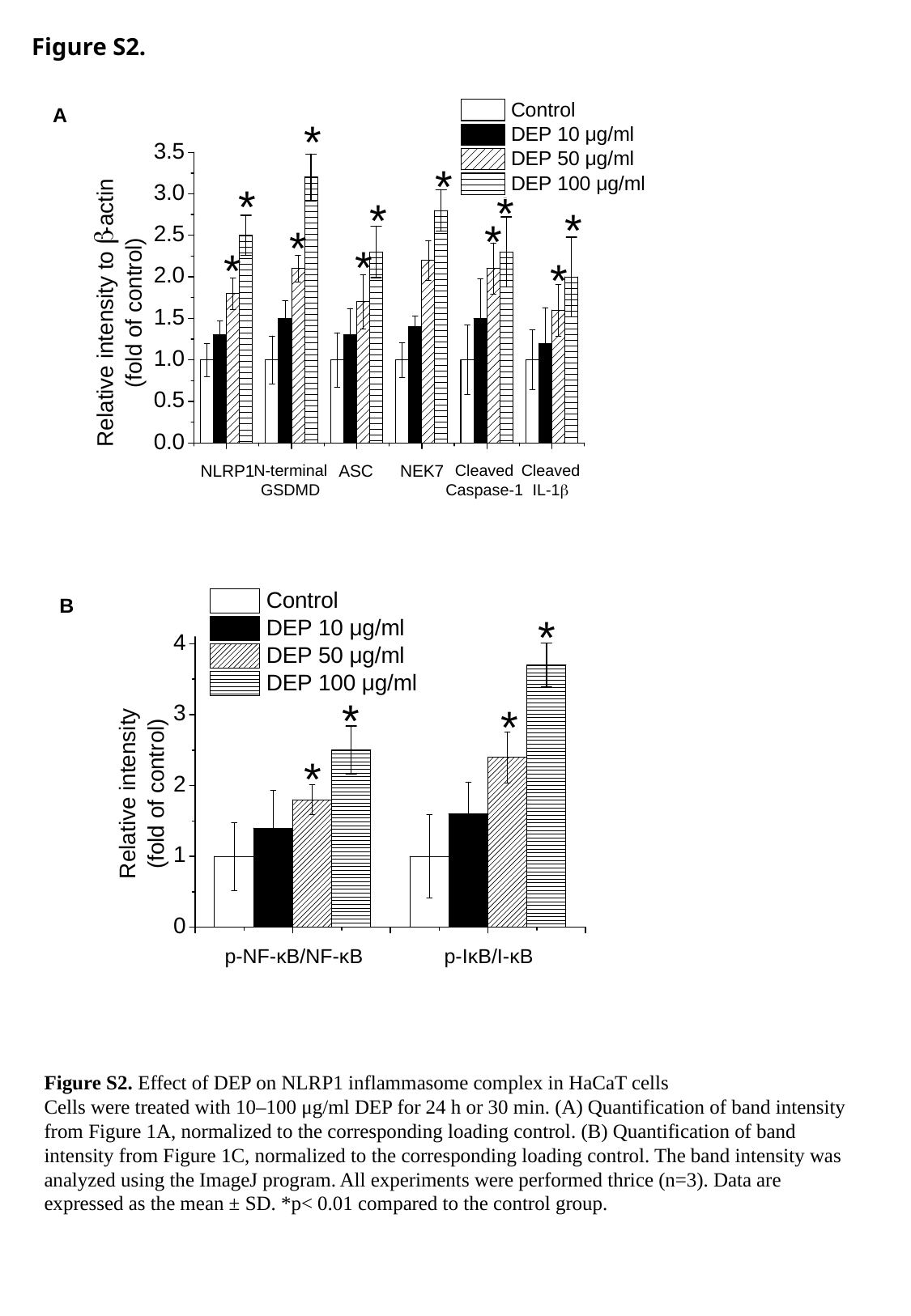

Figure S2.
NLRP1
N-terminal
GSDMD
ASC
NEK7
Cleaved
Caspase-1
Cleaved
IL-1β
A
p-NF-κB/NF-κB
p-IκB/I-κB
B
Figure S2. Effect of DEP on NLRP1 inflammasome complex in HaCaT cells
Cells were treated with 10–100 μg/ml DEP for 24 h or 30 min. (A) Quantification of band intensity from Figure 1A, normalized to the corresponding loading control. (B) Quantification of band intensity from Figure 1C, normalized to the corresponding loading control. The band intensity was analyzed using the ImageJ program. All experiments were performed thrice (n=3). Data are expressed as the mean ± SD. *p< 0.01 compared to the control group.

## Slide 4
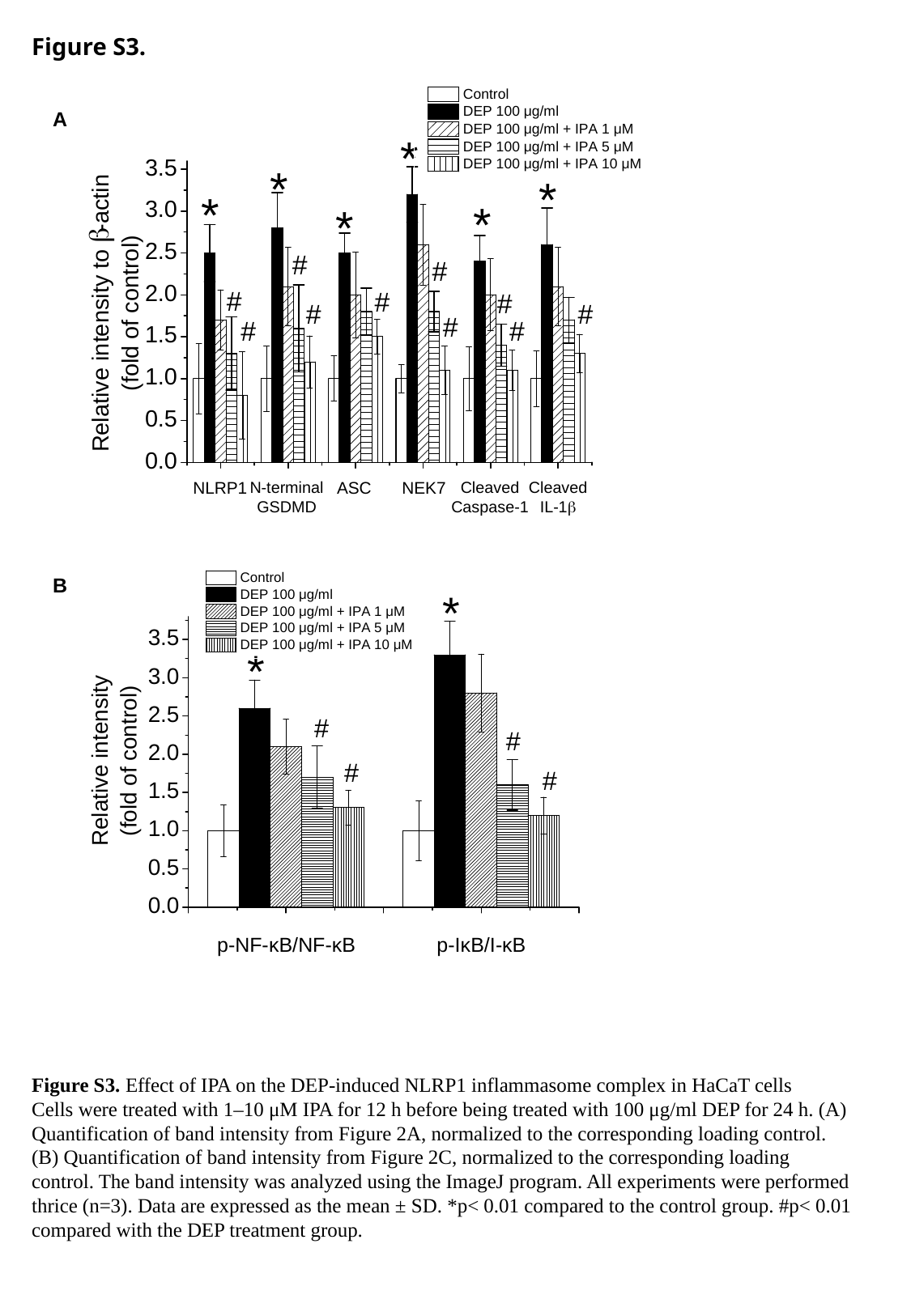

Figure S3.
NLRP1
N-terminal
GSDMD
ASC
NEK7
Cleaved
Caspase-1
Cleaved
IL-1β
A
p-NF-κB/NF-κB
p-IκB/I-κB
B
Figure S3. Effect of IPA on the DEP-induced NLRP1 inflammasome complex in HaCaT cells
Cells were treated with 1–10 μM IPA for 12 h before being treated with 100 μg/ml DEP for 24 h. (A) Quantification of band intensity from Figure 2A, normalized to the corresponding loading control. (B) Quantification of band intensity from Figure 2C, normalized to the corresponding loading control. The band intensity was analyzed using the ImageJ program. All experiments were performed thrice (n=3). Data are expressed as the mean ± SD. *p< 0.01 compared to the control group. #p< 0.01 compared with the DEP treatment group.

## Slide 5
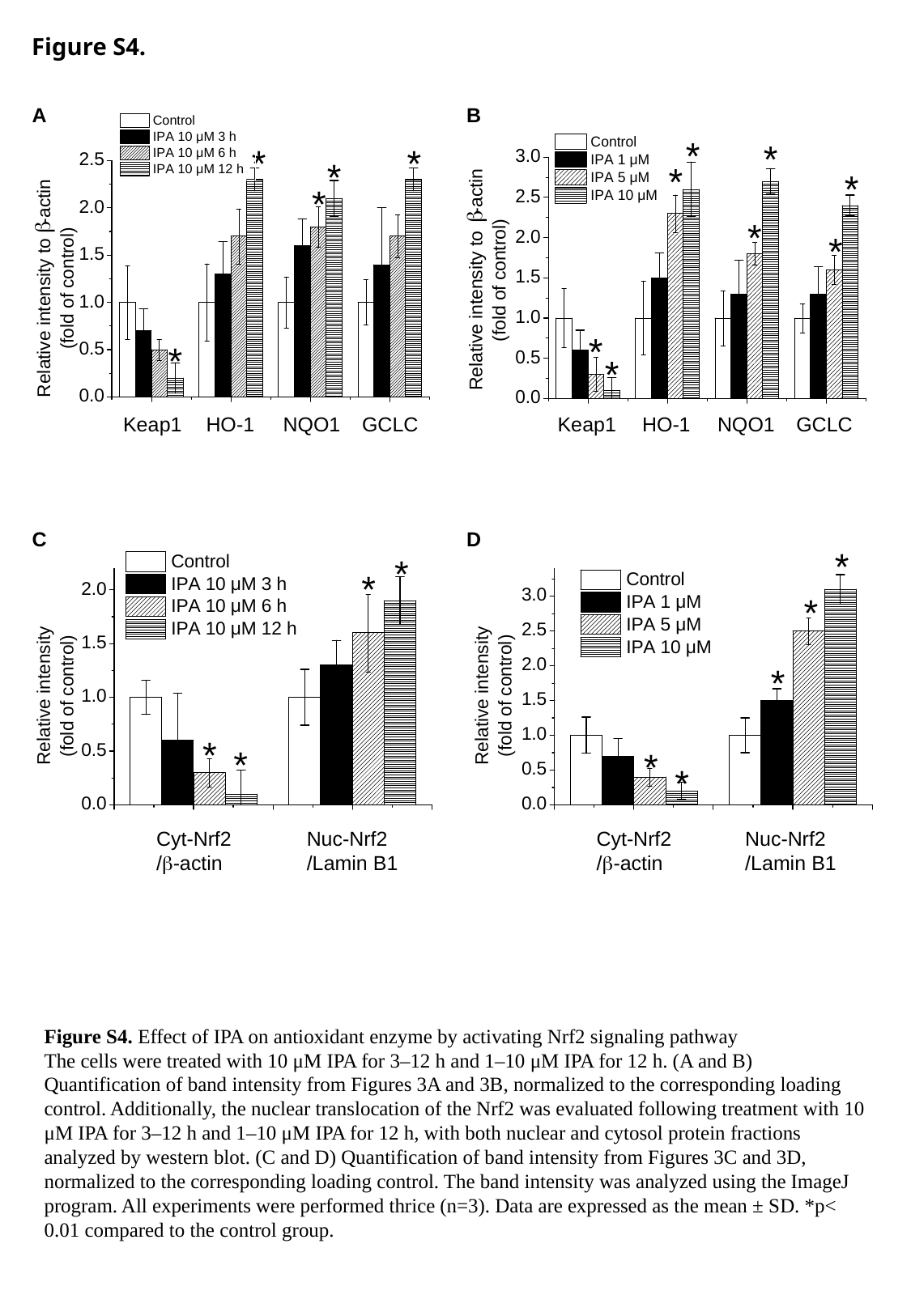

Figure S4.
A
B
Keap1
HO-1
NQO1
GCLC
Keap1
HO-1
NQO1
GCLC
C
D
Cyt-Nrf2
/β-actin
Nuc-Nrf2
/Lamin B1
Cyt-Nrf2
/β-actin
Nuc-Nrf2
/Lamin B1
Figure S4. Effect of IPA on antioxidant enzyme by activating Nrf2 signaling pathway
The cells were treated with 10 μM IPA for 3–12 h and 1–10 μM IPA for 12 h. (A and B) Quantification of band intensity from Figures 3A and 3B, normalized to the corresponding loading control. Additionally, the nuclear translocation of the Nrf2 was evaluated following treatment with 10 μM IPA for 3–12 h and 1–10 μM IPA for 12 h, with both nuclear and cytosol protein fractions analyzed by western blot. (C and D) Quantification of band intensity from Figures 3C and 3D, normalized to the corresponding loading control. The band intensity was analyzed using the ImageJ program. All experiments were performed thrice (n=3). Data are expressed as the mean ± SD. *p< 0.01 compared to the control group.

## Slide 6
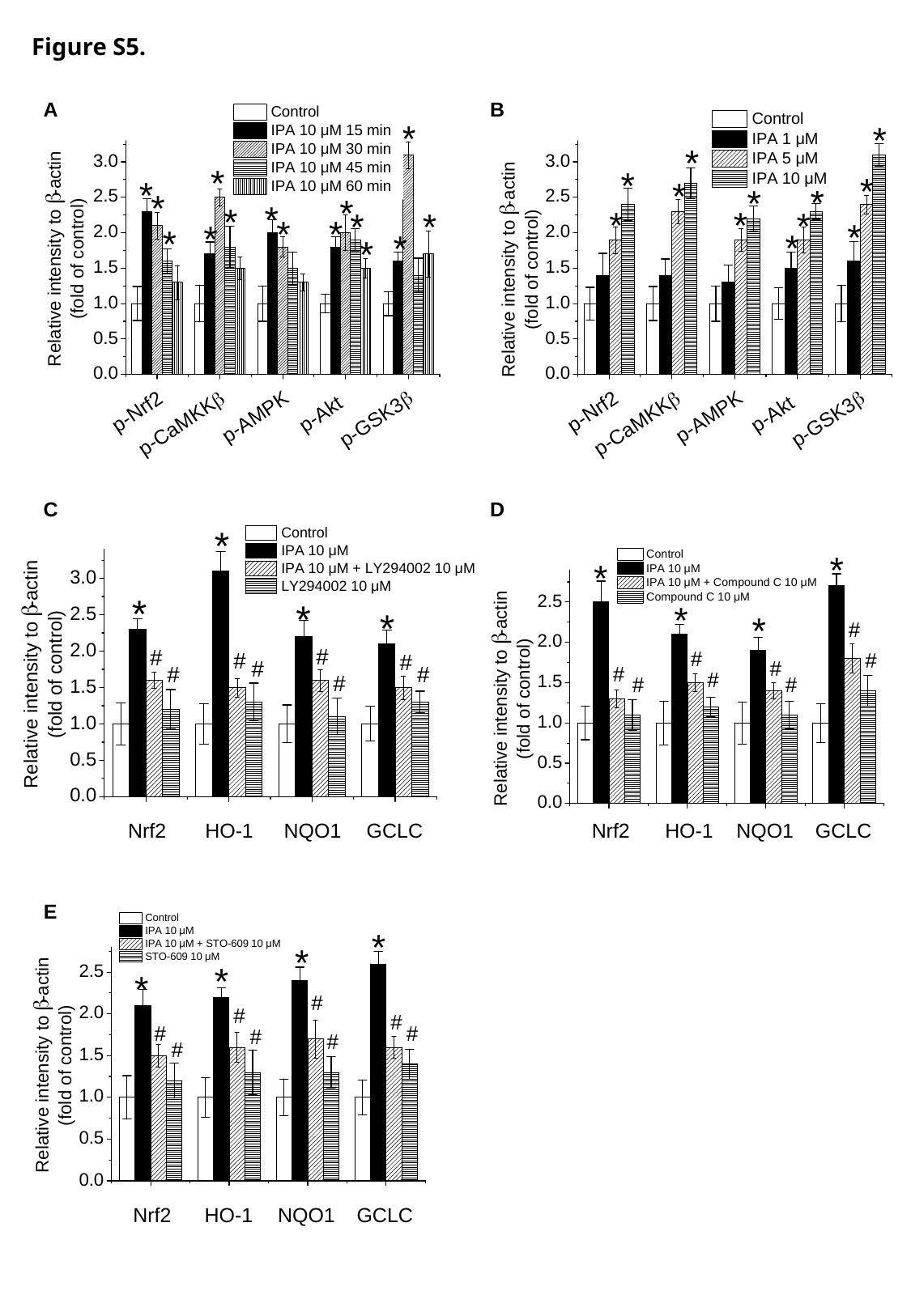

Figure S5.
A
B
p-Nrf2
p-Akt
p-AMPK
p-GSK3β
p-CaMKKβ
p-Nrf2
p-Akt
p-AMPK
p-GSK3β
p-CaMKKβ
C
D
Nrf2
HO-1
NQO1
GCLC
Nrf2
HO-1
NQO1
GCLC
E
Nrf2
HO-1
NQO1
GCLC

## Slide 7
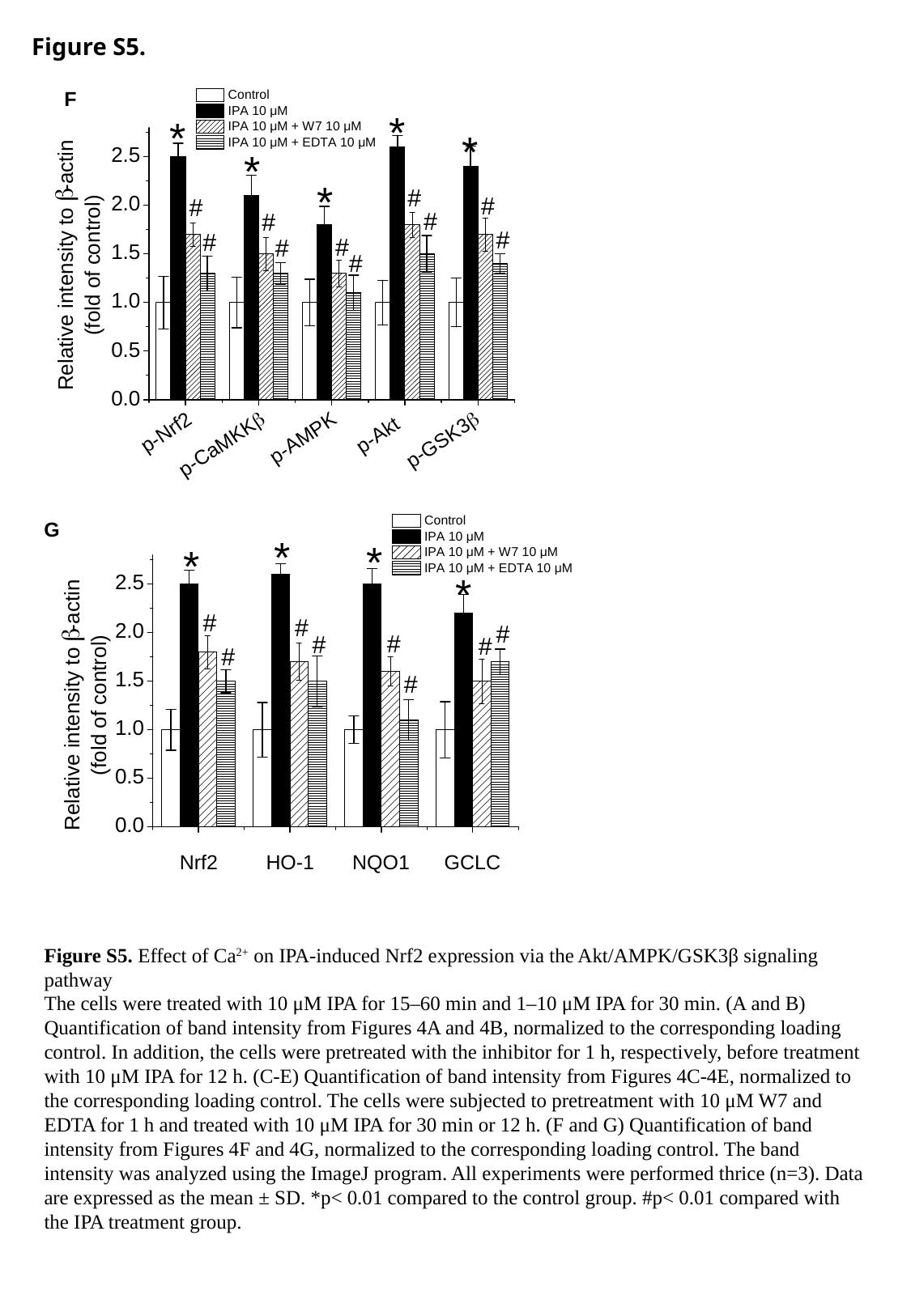

Figure S5.
F
p-Nrf2
p-Akt
p-AMPK
p-GSK3β
p-CaMKKβ
G
Nrf2
HO-1
NQO1
GCLC
Figure S5. Effect of Ca2+ on IPA-induced Nrf2 expression via the Akt/AMPK/GSK3β signaling pathway
The cells were treated with 10 μM IPA for 15–60 min and 1–10 μM IPA for 30 min. (A and B) Quantification of band intensity from Figures 4A and 4B, normalized to the corresponding loading control. In addition, the cells were pretreated with the inhibitor for 1 h, respectively, before treatment with 10 μM IPA for 12 h. (C-E) Quantification of band intensity from Figures 4C-4E, normalized to the corresponding loading control. The cells were subjected to pretreatment with 10 μM W7 and EDTA for 1 h and treated with 10 μM IPA for 30 min or 12 h. (F and G) Quantification of band intensity from Figures 4F and 4G, normalized to the corresponding loading control. The band intensity was analyzed using the ImageJ program. All experiments were performed thrice (n=3). Data are expressed as the mean ± SD. *p< 0.01 compared to the control group. #p< 0.01 compared with the IPA treatment group.

## Slide 8
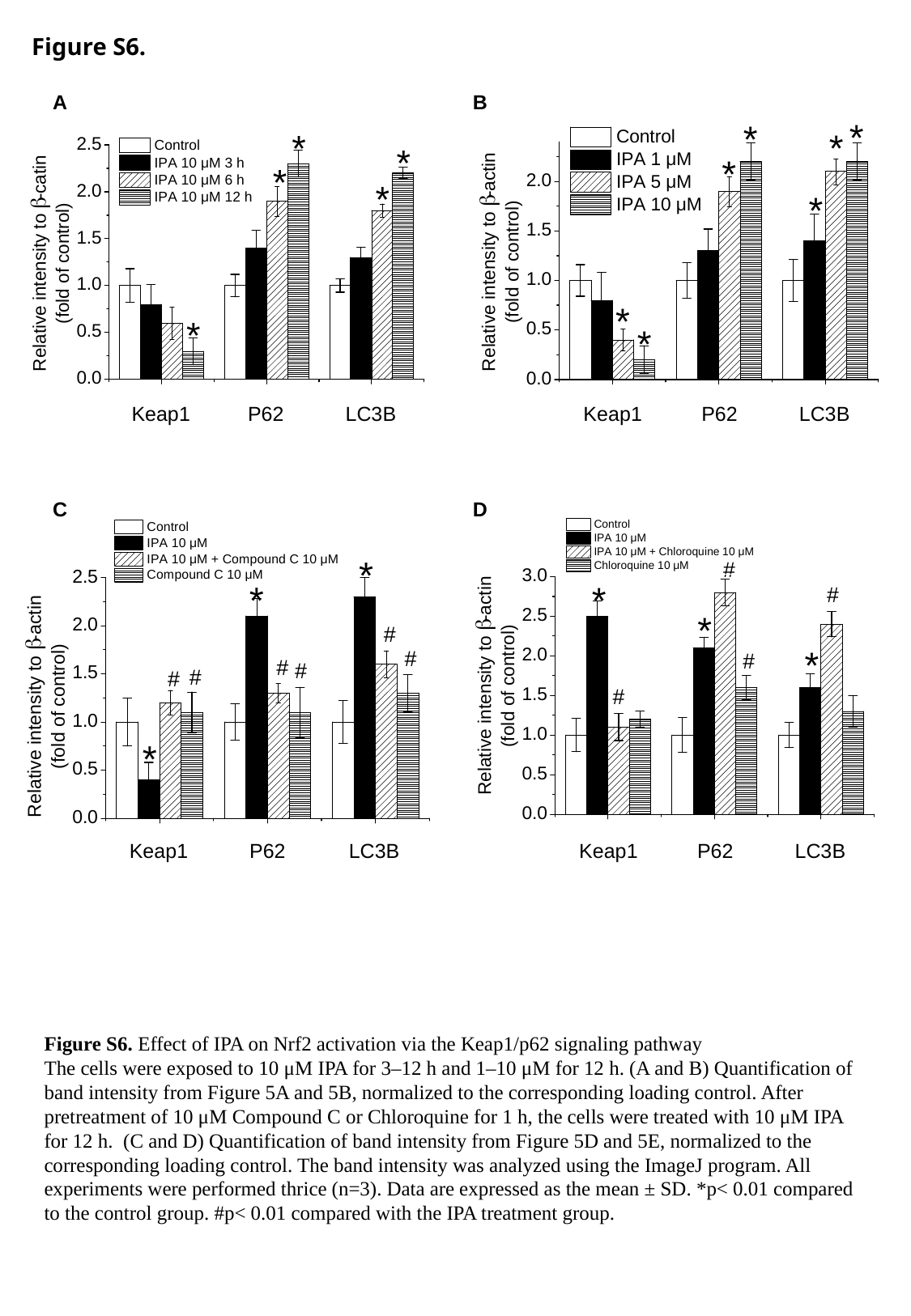

Figure S6.
A
B
Keap1
P62
LC3B
Keap1
P62
LC3B
C
D
Keap1
P62
LC3B
Keap1
P62
LC3B
Figure S6. Effect of IPA on Nrf2 activation via the Keap1/p62 signaling pathway
The cells were exposed to 10 μM IPA for 3–12 h and 1–10 μM for 12 h. (A and B) Quantification of band intensity from Figure 5A and 5B, normalized to the corresponding loading control. After pretreatment of 10 μM Compound C or Chloroquine for 1 h, the cells were treated with 10 μM IPA for 12 h. (C and D) Quantification of band intensity from Figure 5D and 5E, normalized to the corresponding loading control. The band intensity was analyzed using the ImageJ program. All experiments were performed thrice (n=3). Data are expressed as the mean ± SD. *p< 0.01 compared to the control group. #p< 0.01 compared with the IPA treatment group.

## Slide 9
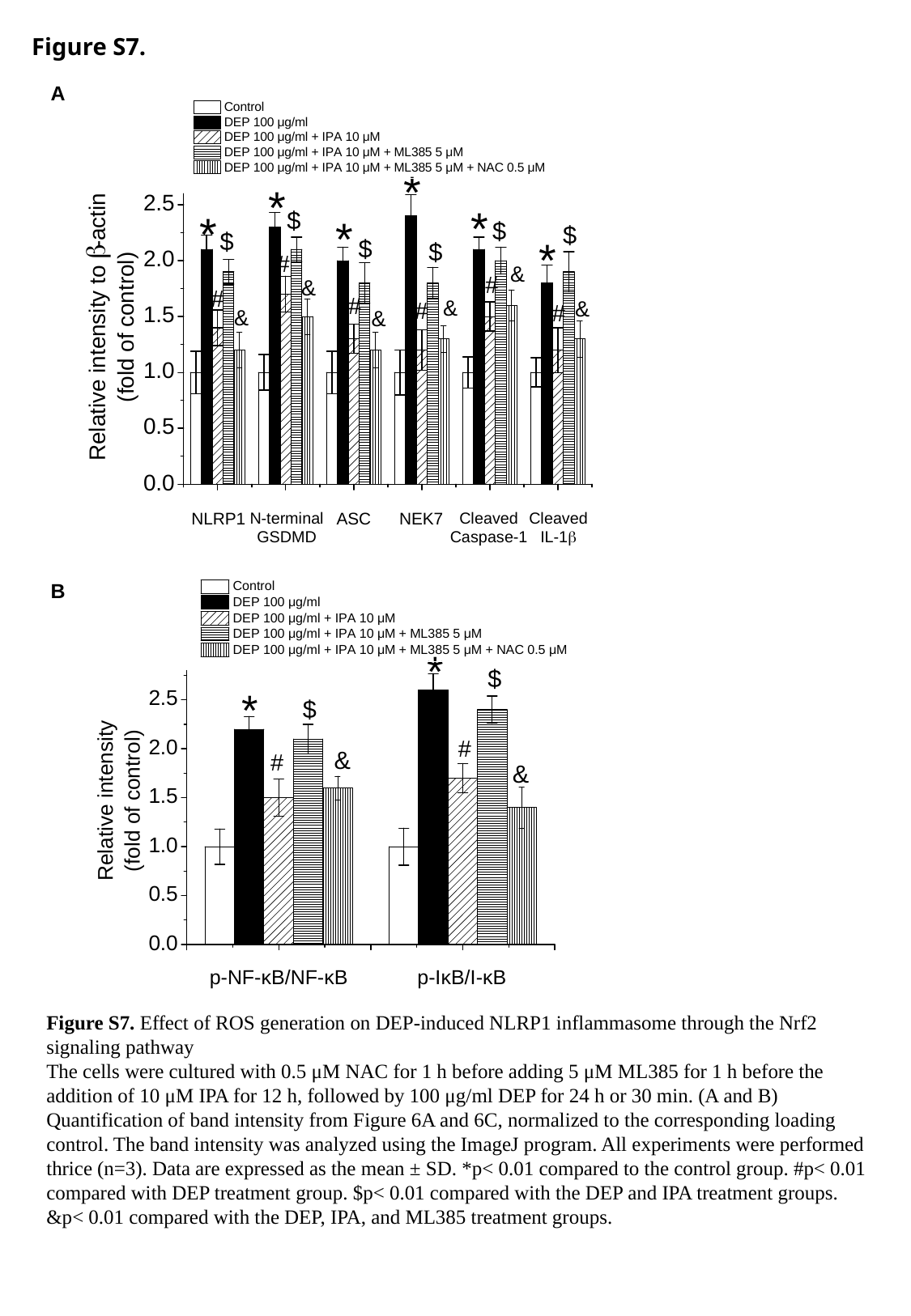

Figure S7.
A
NLRP1
N-terminal
GSDMD
ASC
NEK7
Cleaved
Caspase-1
Cleaved
IL-1β
B
p-NF-κB/NF-κB
p-IκB/I-κB
Figure S7. Effect of ROS generation on DEP-induced NLRP1 inflammasome through the Nrf2 signaling pathway
The cells were cultured with 0.5 μM NAC for 1 h before adding 5 μM ML385 for 1 h before the addition of 10 μM IPA for 12 h, followed by 100 μg/ml DEP for 24 h or 30 min. (A and B) Quantification of band intensity from Figure 6A and 6C, normalized to the corresponding loading control. The band intensity was analyzed using the ImageJ program. All experiments were performed thrice (n=3). Data are expressed as the mean ± SD. *p< 0.01 compared to the control group. #p< 0.01 compared with DEP treatment group. $p< 0.01 compared with the DEP and IPA treatment groups. &p< 0.01 compared with the DEP, IPA, and ML385 treatment groups.
